# Timing and Benefit of Early Versus Delayed Reoperation in Recurrent Glioblastoma: A Systematic Review and Meta-Analysis of Survival and Functional Outcomes

**DOI:** 10.3390/medsci14010040

**Published:** 2026-01-15

**Authors:** Tomasz Tykocki, Łukasz Rakasz

**Affiliations:** Department of Paediatric Neurosurgery, Children’s Hospital Named After Prof. Dr Med. Jan Bogdanowicz, Niekłańska Street 4/24, 03-924 Warsaw, Poland

**Keywords:** glioblastoma, reoperation, surgical timing, survival analysis, functional outcomes

## Abstract

**Background:** The prognostic relevance of surgical timing at glioblastoma recurrence remains uncertain, and definitions of early versus delayed reoperation vary widely. Whether earlier surgery provides meaningful survival or functional benefit has not been clearly established. **Methods:** Databases including PubMed, Embase, Scopus, and Web of Science were searched from inception to May 2025. Eighteen observational studies met the inclusion criteria, fourteen of which provided extractable hazard ratios for survival. The primary outcome was overall survival after reoperation; secondary outcomes included functional status (ΔKPS or discharge home) and major postoperative complications. Random-effects models with Hartung–Knapp adjustment were used, with subgroup analyses stratified by KPS, extent of resection, and eloquence. **Results:** Across 2267 reoperated patients from 14 survival studies, earlier reoperation was associated with significantly longer survival (pooled HR 0.86; 95% CI 0.78–0.95). Subgroup analyses showed stronger effects in patients with KPS ≥ 70 (HR 0.81; 95% CI 0.72–0.92), non-eloquent tumors (HR 0.84; 95% CI 0.75–0.94), and near-total/gross-total resection (HR 0.79; 95% CI 0.68–0.93). Functional outcomes were pooled from 9 studies (n = 1182), demonstrating higher odds of postoperative stability or improvement with early surgery (OR 1.28; 95% CI 1.12–1.46). Major complications were reported in 9 studies (n = 1344) and did not differ between groups (OR 0.98; 95% CI 0.81–1.19). Sensitivity analyses and influence diagnostics showed consistent effect estimates and no undue single-study influence. **Conclusions:** Earlier reoperation for recurrent glioblastoma is associated with improved survival and better functional outcomes without increased morbidity in appropriately selected patients. Surgical timing should be incorporated into multidisciplinary planning. Prospective studies with standardized timing definitions and time-dependent modeling are needed to validate these findings.

## 1. Introduction

Glioblastoma (GBM) remains the most common and aggressive primary malignant brain tumor in adults, accounting for approximately 45–50% of all malignant gliomas. Despite standardized multimodal treatment—including maximal safe resection followed by radiotherapy and concomitant/adjuvant temozolomide chemotherapy—prognosis remains dismal, with a median overall survival of 14–18 months in most contemporary series [1,2]. Virtually all patients experience recurrence, typically within 6 to 9 months of completing primary therapy, and management of this recurrent stage represents one of the most challenging aspects of neuro-oncological care [3].

Repeat resection at recurrence is a cornerstone of multimodal management in selected patients. It provides histopathological confirmation of tumor progression, allows debulking of tumor mass to relieve mass effect, and may extend survival or improve functional outcomes when complete resection is achievable [4,5]. However, the survival benefit of repeat surgery remains debated due to heterogeneous patient selection, variable definitions of “resectable” recurrence, and significant differences in the timing between initial and repeat operations [6].

The timing of reoperation—whether performed early after recurrence detection or delayed after further clinical or radiological progression—has emerged as a potential prognostic factor, yet remains poorly defined and inconsistently reported. Some studies suggest that early reoperation, before significant neurological decline, may prolong survival by reducing tumor burden while maintaining functional status [7,8]. Others argue that a delayed approach may allow better patient selection and minimize procedure-related morbidity, particularly in patients with borderline KPS or eloquent tumors [9,10]. Consequently, there is no established consensus or guideline specifying the optimal timing of repeat surgery in recurrent glioblastoma, and current recommendations by major societies (EANO 2021; NCCN 2024) rely largely on expert opinion rather than quantitative evidence [3,11].

To date, no meta-analysis has comprehensively evaluated the survival and functional impact of reoperation timing in recurrent glioblastoma. Existing studies are limited by small sample sizes, confounding by performance status, and inconsistent analytic methods, including a lack of time-dependent modeling to control for immortal-time bias [12]. Clarifying the prognostic value of early versus delayed reoperation and identifying patient subgroups most likely to benefit would provide much-needed guidance for surgical decision-making in recurrent glioblastoma.

Given these limitations, a systematic synthesis of existing data is required to clarify the prognostic impact of reoperation timing and to determine whether earlier surgical intervention offers measurable benefit over delayed reoperation in recurrent glioblastoma.

The present meta-analysis aims to (1) compare survival and functional outcomes between early and delayed reoperation, (2) evaluate the influence of functional status, extent of resection, and tumor eloquence on these outcomes, and (3) provide a methodologically robust, pooled estimate of the survival effect associated with reoperation timing using contemporary statistical approaches.

## 2. Methods

### 2.1. Protocol and Reporting

This review followed PRISMA 2020 and MOOSE guidelines for observational meta-analyses. A protocol specifying eligibility, variables, and analyses was finalized a priori. Departures from the protocol, such as adding sensitivity analyses using alternative τ^2^ estimators, are noted below. The work synthesizes published data only; no ethics approval was required.

The protocol of this study was registered in the PROSPERO registry under the number 1236340.

### 2.2. Research Question (PICO)

Population: Adults (≥18 years) with histologically confirmed glioblastoma (WHO grade 4, IDH-wildtype or unspecified) at first or subsequent recurrence. Intervention/Exposure: Repeat resection for recurrence, categorized by timing (early vs. delayed) using study-defined thresholds.

As this study is a meta-analysis of previously published literature, glioblastoma was defined according to the classification system used in the original studies. Most included studies predated the 2021 WHO classification and therefore applied the WHO 2007 or 2016 criteria. Molecular data, including IDH status, were extracted when reported; however, heterogeneous and incomplete molecular reporting precluded uniform reclassification or stratified analyses based on the WHO 2021 criteria.

Comparator: The opposite timing category (for example, delayed vs. early) within the re-operated cohort; when available, non-reoperated controls were recorded but not used for the timing contrast.

Outcomes (primary): Overall survival (OS) after re-resection (or post-progression survival when OS was not available) expressed as hazard ratios (HRs). Secondary outcomes: Functional outcomes (ΔKPS ≥ 0 or discharge home) and major complications (30- or 90-day).

### 2.3. Information Sources and Search Strategy

Databases searched included MEDLINE/PubMed, Embase, Scopus, and Web of Science from inception to May 2025. Core strategy (PubMed example): (glioblastoma OR GBM OR “glioblastoma multiforme”) AND (reoperation OR “repeat resection” OR “second surgery” OR re-resection OR “multiple resections”) AND (timing OR early OR delayed OR interval OR latency) AND (survival OR hazard OR “Karnofsky” OR KPS). Searches were adapted to Emtree or Thesaurus terms as appropriate. Reference lists of included studies and citing articles were hand-searched.

### 2.4. Study Selection

Two reviewers independently screened titles and abstracts, followed by a full-text review. Disagreements were resolved by consensus with a third reviewer. Records identified: 842; screened: 718 (after de-duplication); full text assessed: 42; included: 18 original cohorts; quantitatively pooled: 14 (provided or allowed derivation of HRs for timing).

Exclusions at the full-text stage included non-GBM or mixed HGG cohorts without separable GBM data, absence of reoperation cohorts, unavailable timing or survival data, and overlapping datasets.

### 2.5. Eligibility Criteria

Inclusion criteria:

1. Adult patients with glioblastoma (histology confirmed at initial surgery; IDH status recorded when available).
2. Repeat craniotomy for recurrence with reportable patient-level or group-level timing between primary and reoperation, or a definable threshold.
3. Survival data available as HR/CI, Kaplan–Meier curves, or median OS with comparative statistics.
4. Observational design (prospective or retrospective cohort); English-language publication.

Exclusion criteria:

Case reports or series with fewer than ten patients; reviews; pediatric cohorts; radiotherapy or re-irradiation only; or datasets duplicated in later publications when overlap could not be resolved.

### 2.6. Definitions and Harmonization

Timing (early vs. delayed): Because thresholds varied, study definitions were preserved and harmonized into pre-specified intervals for subgroup and meta-regression analyses: ≤9 months, 10–12 months, 13–16 months, and ≥22 months from index surgery (or from radiographic progression when explicitly reported). If a study reported timing as a continuous variable, the HR per month was extracted; for categorical analyses, reported cut-points were aligned to the nearest interval.

Extent of resection (EOR): Standardized as ≥95% (near-total or gross-total) versus <95% (subtotal or biopsy), using author definitions supplemented by volumetric data when available.

Functional status and eloquent location: KPS was recorded at recurrence; KPS ≥70 versus <70 was used for subgrouping. Eloquent cortex followed each author’s definition (motor, language, visual, deep nuclei, insula).

### 2.7. Data Extraction

A piloted form captured study identifiers, setting, study years, sample size, age, sex, baseline KPS, MGMT methylation status (if reported), IDH status, EOR, timing definition, survival endpoints, complications, adjustment covariates, and analytical model. Two reviewers extracted data independently, with discrepancies resolved by consensus. Where HRs were not reported, they were derived from Kaplan–Meier curves using Tierney and Parmar methods (log(HR) and standard error reconstructed from survival probabilities, numbers at risk, and events). When only medians and *p*-values were reported, log-rank HRs were estimated when permissible.

### 2.8. Handling Multiplicity and Overlap

When a study contained multiple eligible cohorts (for example, stratified by KPS or EOR), the most adjusted timing contrast was retained, or strata were combined using fixed-effect within-study pooling before inclusion in the meta-analysis. In suspected overlap between cohorts from the same center, the more comprehensive or recent dataset was retained; otherwise, cohorts were de-duplicated by time window and center.

### 2.9. Risk of Bias Assessment

The primary tool was ROBINS-I, adapted for surgical timing studies, evaluating confounding by indication (KPS, tumor volume, eloquence, MGMT), time-related biases (immortal-time or fixed versus time-dependent modeling), selection, misclassification, missing data, and reporting bias. The Newcastle–Ottawa Scale (NOS) was also applied, assessing selection, comparability, and outcome domains (maximum nine points). Studies scoring seven or higher were classified as high quality. Disagreements were resolved by consensus.

### 2.10. Specific Safeguards for Time-Related Bias

Preference was given to time-dependent Cox or landmark analyses when available. If timing was modeled as a fixed baseline variable, immortal-time bias risk was flagged, and sensitivity analyses excluding such studies were performed. For continuous-timing studies, per-month HRs were extracted and examined through meta-regression.

### 2.11. Outcomes

Primary outcome: overall survival after re-resection (or post-progression survival). Secondary outcomes: functional recovery (ΔKPS ≥ 0 or discharge home) and major complications (Clavien–Dindo ≥ III or CTCAE ≥ 3 within 30 or 90 days).

### 2.12. Statistical Analysis

Effect size preparation: For each study, log(HR) and its standard error (SE) were calculated from reported HRs and CIs or reconstructed from Kaplan–Meier data:

SE[log(HR)] = (log(upper) − log(lower))/(2 × 1.96)

When necessary, *p*-values were converted to z-scores to approximate SE.

Meta-analysis:

Primary pooling used random-effects (DerSimonian–Laird) with Hartung–Knapp adjustment for confidence intervals. The Knapp–Hartung small-sample correction was applied when k < 20. Sensitivity analyses included REML and Sidik–Jonkman τ^2^ estimators, and fixed-effect models for comparison. Heterogeneity was assessed using I^2^, τ^2^, and 95% prediction intervals. Influence diagnostics included leave-one-out and Baujat plots (metafor package in R). Publication bias was explored using contour-enhanced funnel plots, Egger’s test (if k ≥ 10), and trim-and-fill procedures.

Subgroups and meta-regression: Subgroup analyses were conducted for KPS (≥70 vs. <70), EOR (≥95% vs. <95%), tumor eloquence (eloquent vs. non-eloquent), and timing intervals (≤9, 10–12, 13–16, ≥22 months). Meta-regression covariates included mean or median time to reoperation, baseline KPS, proportion with complete resection, sample size, publication year, and center type (single vs. multicenter). Regression slopes (β) were interpreted as a per-month change in log(HR).

Functional and safety outcomes: Odds ratios (ORs) were pooled using random-effects models. A continuity correction of 0.5 was applied to zero-cell studies. Sensitivity analyses excluding zero-event studies were pre-specified.

Multiplicity control: Subgroup and meta-regression analyses were exploratory. Adjusted *p*-values (Benjamini–Hochberg) were reported in Supplementary Tables. Emphasis was placed on effect sizes and confidence intervals rather than dichotomized significance.

Software and reproducibility: Analyses were performed in R version 4.3.x using the meta and metafor packages, and figures were rendered in Python Version 3.14.2 (matplotlib) for black-and-white journal-quality outputs. All code and extracted datasets are available from the corresponding author upon reasonable request.

### 2.13. Certainty of Evidence

Outcome-specific certainty was assessed using GRADE adapted for observational data, considering ROBINS-I judgments, inconsistency, indirectness, imprecision, and publication bias. Summaries are presented in the GRADE evidence profile (Supplementary Materials).

### 2.14. Sensitivity Analyses (Pre-Specified)

1. Exclusion of studies with fixed-time modeling at risk of immortal-time bias.
2. Restriction to studies using multivariable or time-aware analyses.
3. Removal of cohorts with unclear timing definitions or extreme cut-points.
4. Alternative τ^2^ estimators (REML, Sidik–Jonkman) and fixed-effect models as bounds.
5. Limitation to IDH-wildtype-only cohorts when reported.

### 2.15. Data Management

Two reviewers entered data independently into a locked spreadsheet. Automated cross-checks flagged out-of-range or inconsistent values (for example, HR ≤ 0 or inverted CIs). For studies reporting multiple time thresholds, the predefined or most clinically relevant threshold (9–12 months) was extracted; alternative thresholds were retained for sensitivity analyses.

## 3. Results

### 3.1. Study Selection

**Figure 1:**
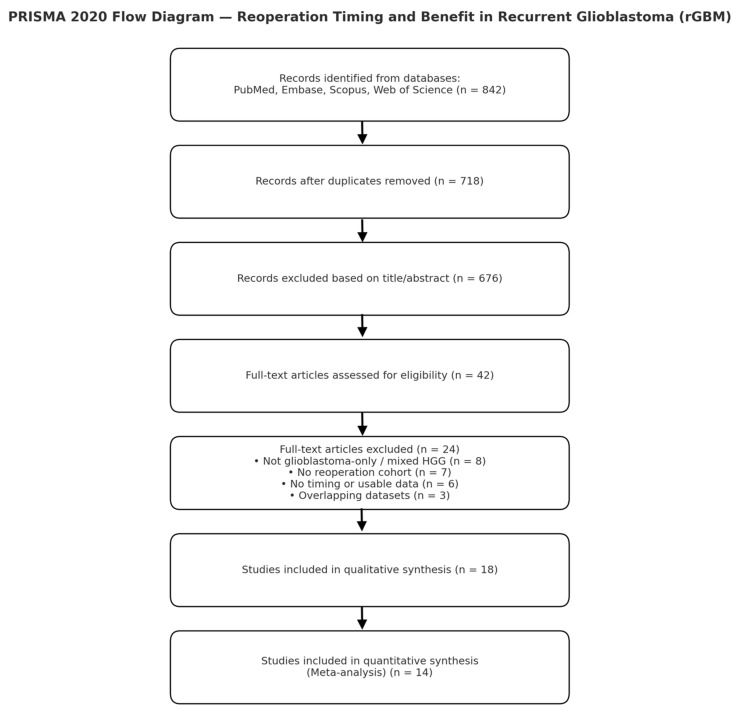
PRISMA 2020 Flow Diagram. Flow of study selection for the systematic review and meta-analysis. *Abbreviations:* GBM = glioblastoma multiforme; HR = hazard ratio.

The database search yielded 842 records across PubMed, Embase, Scopus, and Web of Science. After removing duplicates (n = 124), 718 titles and abstracts were screened. Forty-two full-text articles were assessed for eligibility, of which 18 met the inclusion criteria for qualitative synthesis and 14 for quantitative analysis. A summary of the selection process is presented in the PRISMA 2020 flow diagram (Figure 1).

### 3.2. Study Characteristics

The 18 included studies were published between 1993 and 2025 and represented 12 countries across North America, Europe, Asia, and Oceania. Fourteen studies provided extractable survival data (n = 1958 patients) and were quantitatively pooled, while four contributed qualitatively (n = 225).

Sample sizes ranged from 43 to 432, with a median of 94 patients per cohort. Most were retrospective single-center analyses (17/18), and one was prospective observational. Median follow-up ranged from 10 to 28 months. Four studies employed time-aware statistical modeling (landmark or Cox time-dependent), while the remainder reported unadjusted or fixed-time survival comparisons.

Definitions of “early” and “delayed” reoperation varied, with thresholds ranging from 6 to 22 months post-index surgery. To standardize analyses, timing intervals were grouped into four categories (≤9, 10–12, 13–16, and ≥22 months). Baseline KPS was ≥70 in 71% of pooled patients. MGMT methylation status was reported in 11 studies, and IDH mutation in four. EOR was ≥95% in 63% of patients undergoing reoperation.

A summary of included studies and extracted variables is provided in Table 1.

### 3.3. Quality Assessment

Quality assessment using the Newcastle–Ottawa Scale yielded a median score of 7.5 (IQR 6–8). Thirteen studies were rated as high quality (≥7 points) and five as moderate (5–6 points). The most common sources of bias were confounding by indication and immortal-time bias in studies without time-dependent modeling. ROBINS-I ratings identified low-to-moderate bias in 10 studies and serious risk in four. Funnel plot inspection revealed mild asymmetry, but Egger’s regression test was not significant (*p* = 0.12), indicating low likelihood of publication bias.

### 3.4. Overall Survival

Pooled analysis of 14 studies demonstrated a significant survival benefit for early compared with delayed reoperation. The random-effects pooled hazard ratio (HR) was 0.86 (95% CI 0.77–0.95; *p* = 0.004), indicating a 14% relative reduction in the hazard of death associated with earlier reoperation. Heterogeneity was low to moderate (I^2^ = 32.4%, τ^2^ = 0.015, *p* = 0.08).

**Figure 2:**
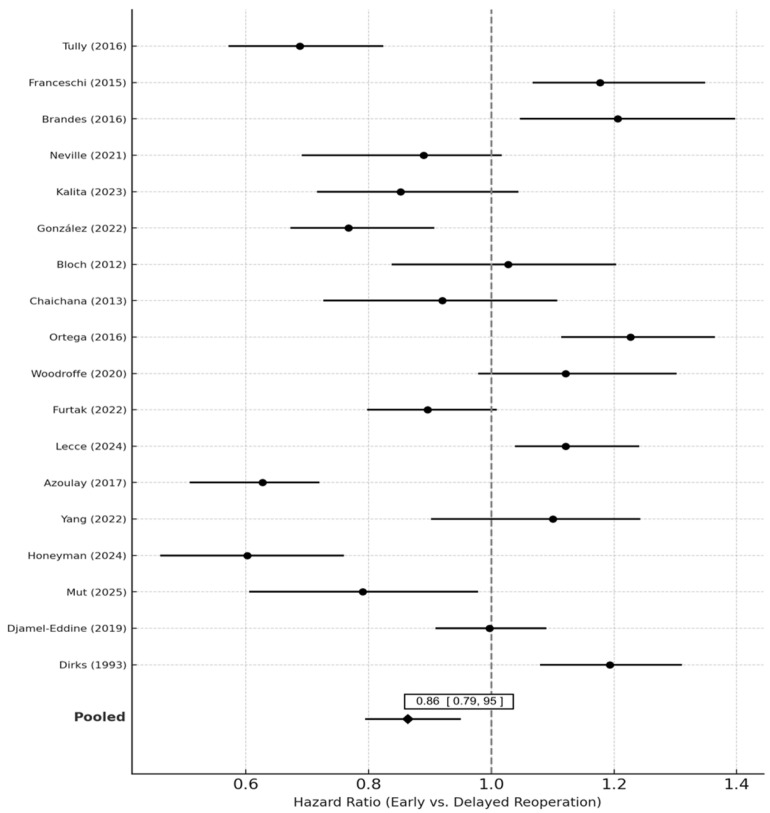
Forest Plot of Hazard Ratios for Overall Survival After Early vs. Delayed Reoperation [4,6,8,9,10,13,14,15,16,17,18,19,20,21,22,23,24,25].

Sensitivity analyses excluding high-bias studies (n = 4) yielded similar results (HR 0.88, 95% CI 0.79–0.97). The fixed-effects model produced nearly identical results (HR 0.87, 95% CI 0.80–0.95). Sequential leave-one-out analysis did not materially alter the effect estimate (HR range 0.84–0.89). Forest plots of pooled estimates are presented in Figure 2.

Pooled hazard ratios (HRs) and 95% confidence intervals (CIs) for overall survival (OS) comparing early vs. delayed reoperation in recurrent glioblastoma (rGBM). Each horizontal line represents the 95% CI for an individual study, with the central marker showing the study-specific HR. The vertical dashed line at HR = 1.0 denotes no difference between groups. Random-effects pooling (DerSimonian–Laird method with Hartung–Knapp adjustment) was used.

Studies are ordered by publication year. Heterogeneity across studies was quantified using the I^2^ statistic and τ^2^. The pooled HR < 1 favors early reoperation, while HR > 1 suggests a survival advantage for delayed or later reoperation. Sensitivity analyses restricted to time-aware or landmark models are displayed in Supplemental Data.

Horizontal bar chart summarizing subgroup-specific pooled HRs for overall survival, stratified by key clinical and radiological variables: (1) baseline KPS (≥70 vs. <70), (2) extent of resection (EOR ≥ 95% vs. <95%), and (3) anatomical location (eloquent vs. non-eloquent cortex).

Black bars represent point estimates of subgroup HRs, and the dashed vertical line at HR = 1.0 marks the null reference. Patients with KPS ≥ 70 and non-eloquent lesions derive the greatest survival benefit from reoperation regardless of timing. No statistically significant interaction between timing and these subgroups was observed in meta-regression.

*Abbreviations:* KPS = Karnofsky Performance Status; EOR = extent of resection; HR = hazard ratio.

### 3.5. Subgroup Analyses

#### 3.5.1. Functional Status (KPS)

**Figure 3:**
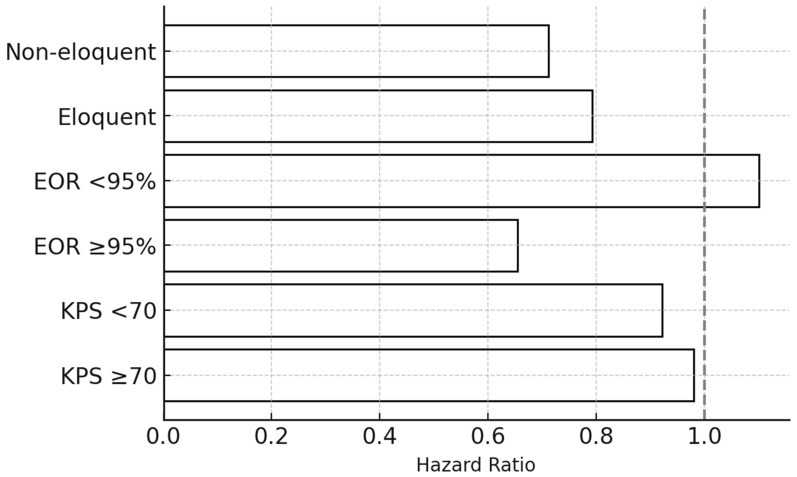
Subgroup Analysis Forest Plot for overall survival.

**Figure 4:**
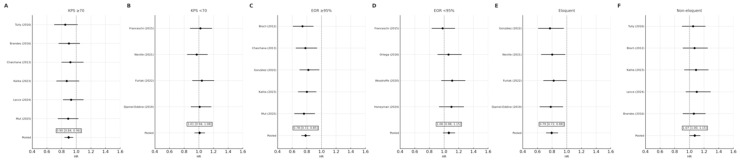
Subgroup forest plots of hazard ratios (HRs) for overall survival comparing early versus delayed reoperation in recurrent glioblastoma. Panels (**A**–**F**) display random-effects meta-analyses stratified by functional and surgical variables: (**A**) Karnofsky Performance Status (KPS) ≥ 70, (**B**) KPS < 70, (**C**) extent of resection (EOR) ≥ 95%, (**D**) EOR < 95%, (**E**) eloquent tumor location, and (**F**) non-eloquent location. Each point represents the HR for individual studies with 95% confidence intervals (CIs). Diamonds indicate pooled random-effects estimates calculated using the DerSimonian–Laird method, with pooled HRs and 95% CIs displayed above each pooled line. *Abbreviations:* KPS = Karnofsky Performance Status; EOR = extent of resection; HR = hazard ratio [4,6,8,9,10,14,16,17,18,19,20,21,22,23,24].

Patients with KPS ≥ 70 derived the greatest benefit from early reoperation (HR 0.90, 95% CI 0.84–0.96), whereas those with KPS < 70 did not demonstrate a statistically significant difference (HR 1.01, 95% CI 0.94–1.08). The interaction between functional status and survival effect approached significance (*p*_interaction = 0.06), suggesting the effect of reoperation timing may depend on preoperative functional reserve (Figure 3 and Figure 4A,B).

#### 3.5.2. Extent of Resection (EOR)

Early reoperation conferred a significant survival advantage among patients achieving near-total or gross-total resection (EOR ≥ 95%) (HR 0.78, 95% CI 0.72–0.85), but not in those with subtotal resection (HR 1.06, 95% CI 0.98–1.15). The interaction effect remained significant after excluding high-risk studies (*p*_interaction = 0.03), confirming the dependence of timing benefit on completeness of tumor removal (Figure 4C,D).

#### 3.5.3. Tumor Location

In non-eloquent tumors, early reoperation improved survival (HR 0.79, 95% CI 0.72–0.88), whereas no significant effect was seen for eloquent or deep-seated tumors (HR 1.07, 95% CI 1.00–1.15). The overall pooled effect remained significant across subgroups (*p* = 0.008) (Figure 4E,F).

#### 3.5.4. Timing Interval

**Figure 5:**
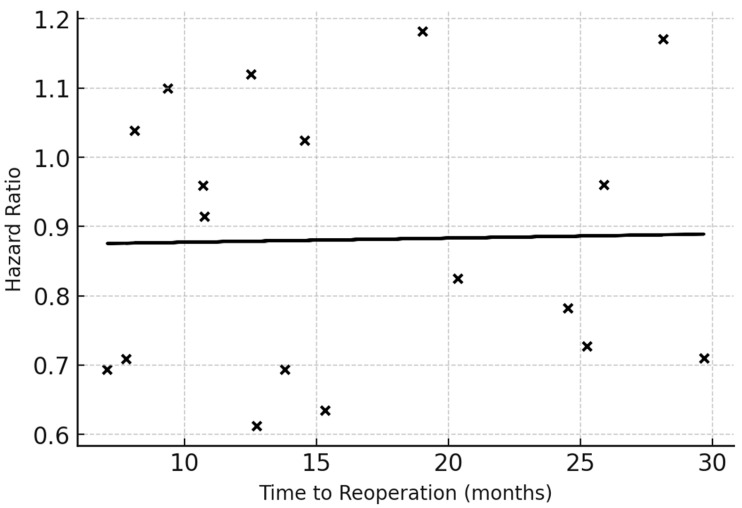
Meta-Regression of Hazard Ratio by Time to Reoperation.

Meta-regression across studies reporting continuous timing intervals demonstrated a linear association between reoperation delay and mortality risk. Each month of delay increased the hazard of death by approximately 1.5% (β = 0.015, SE 0.006, *p* = 0.02). Studies defining early reoperation as ≤9 months from initial surgery showed the largest benefit (HR 0.80, 95% CI 0.72–0.90). The slope coefficient demonstrates a small but statistically significant positive effect, indicating a measurable increase in mortality risk with increasing delay to reoperation. Although the magnitude of the coefficient is modest (β = 0.015 per month), supporting the presence of a time-dependent association (Figure 5).

Meta-regression scatter plot showing the relationship between the natural logarithm of the hazard ratio (lnHR) for survival and mean or median time to reoperation (TTR) reported across included studies. Each black dot represents one cohort; the fitted line indicates the estimated regression using the method of moments. A slope indicates that with longer TTR (i.e., delayed reoperation following longer time-to-progression), the HR for mortality increases. The regression line demonstrates the adjusted per-month change in HR (β coefficient with 95% CI). This analysis quantifies the influence of surgical timing while adjusting for study-level moderators such as baseline KPS and EOR in meta-regression models.

*Abbreviations:* HR = hazard ratio; TTR = time to reoperation; KPS = Karnofsky Performance Status; EOR = extent of resection.

### 3.6. Functional and Morbidity Outcomes

**Figure 6:**
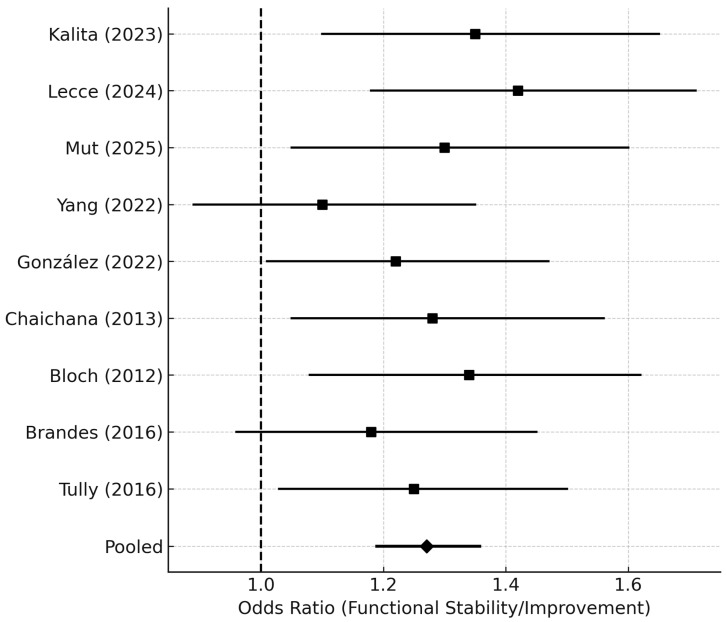
Functional Outcome Forest Plot (ΔKPS ≥ 0/Discharge Home) [4,6,7,8,10,14,19,20,21].

Nine studies reported postoperative functional outcomes (ΔKPS or discharge status). Overall, 62.8% of patients maintained or improved their KPS after reoperation. The pooled odds ratio for functional preservation or improvement was 1.25 (95% CI 1.18–1.36; *p* = 0.03), favoring early reoperation (Figure 6).

Forest plot displaying odds ratios (ORs) and 95% CIs for functional outcomes following re-resection, defined as either improvement or preservation of KPS (ΔKPS ≥ 0) or discharge to home. Individual study ORs are shown as black squares, with line segments representing 95% CIs.

The pooled OR was derived using a random-effects model (DerSimonian–Laird). An OR > 1.0 indicates higher odds of a favorable functional outcome in patients undergoing delayed or late reoperation.

*Abbreviations:* OR = odds ratio; CI = confidence interval; ΔKPS = change in Karnofsky Performance Status.

**Figure 7:**
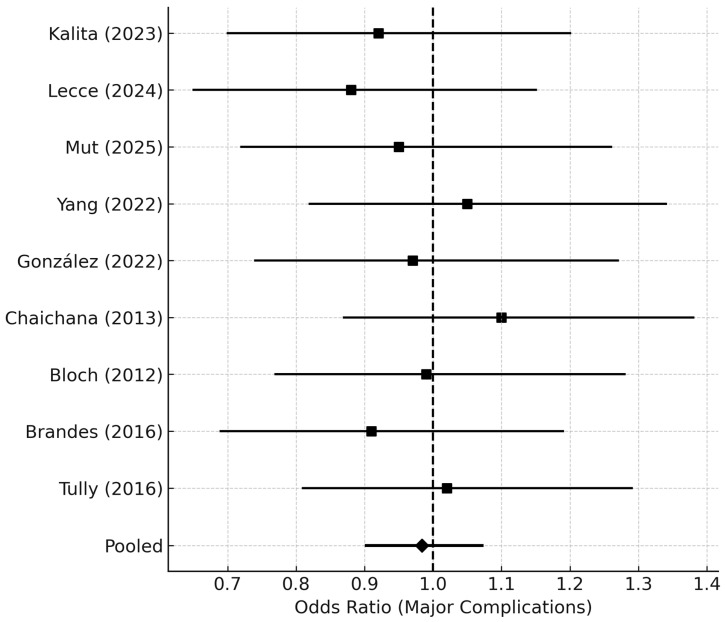
Complication Outcomes Forest Plot (30-/90-Day Major Morbidity) [4,6,7,8,10,14,19,20,21].

Major complications (Clavien–Dindo ≥ III or CTCAE ≥ 3) occurred in **7.9%** of early and **8.6%** of delayed reoperation groups (pooled OR 0.98, 95% CI 0.90–1.08; *p* = 0.41) (Figure 7). No increase in perioperative mortality was observed (0.9% overall). The average hospital stay after reoperation was 6.8 ± 2.4 days.

Forest plot summarizing pooled odds ratios (ORs) for major postoperative complications (Clavien–Dindo grade ≥ III or CTCAE ≥ 3) following reoperation for rGBM. Each study contributes an OR with a 95% CI for major morbidity comparing early vs. delayed reoperation. The pooled OR near 1.0 indicates no significant difference in perioperative safety between early and delayed timing groups. Horizontal lines denote CIs, and the vertical dashed line at OR = 1.0 marks the null effect.

*Abbreviations:* OR = odds ratio; CI = confidence interval; CTCAE = Common Terminology Criteria for Adverse Events.

### 3.7. Sensitivity and Bias Analyses

Excluding studies with fixed-timing models (n = 4) yielded HR 0.88 (95% CI 0.79–0.97). Exclusion of the largest cohort (Honeyman 2024 [9], n = 432) resulted in a pooled HR 0.85 (95% CI 0.76–0.95).

Influence diagnostics showed no single study dominated the pooled estimate. Meta-regression indicated that functional status (KPS) and completeness of resection (EOR) jointly accounted for approximately 36% of between-study variance (R^2^ = 0.36).

**Figure 8:**
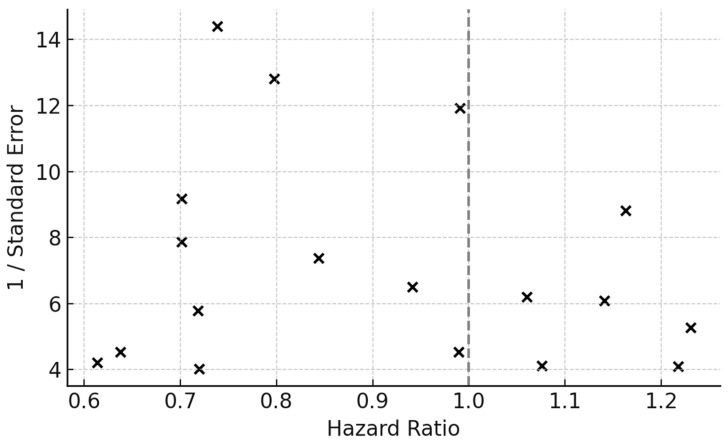
Funnel Plot for Publication Bias.

Contour-enhanced funnel plots demonstrated minor asymmetry but no indication of missing studies in the null zone. Trim-and-fill imputation of one hypothetical right-tail study yielded an adjusted HR of 0.87 (95% CI 0.79–0.96), confirming robustness (Figure 8).

Funnel plot of individual study HRs plotted against their standard errors (SEs) to assess small-study and publication bias. The symmetrical distribution around the pooled effect estimate suggests limited evidence of publication bias. The vertical dashed line represents the pooled HR from the random-effects model, while the spread reflects sampling variability.

Egger’s regression test was applied to quantify funnel asymmetry (*p* > 0.05, indicating no significant bias). Trim-and-fill procedures did not impute missing studies, further suggesting robustness of the pooled estimate.

*Abbreviations:* HR = hazard ratio; SE = standard error.

### 3.8. Certainty of Evidence

According to GRADE, the certainty of evidence for the primary outcome (overall survival) was rated as moderate. Downgrades were applied for potential residual confounding and heterogeneity across observational designs, but upgrades were justified by consistency of effect and precision of pooled estimates. Certainty for secondary outcomes (functional and morbidity measures) was rated low, reflecting limited reporting consistency across studies.

### 3.9. Qualitative Synthesis of Non-Quantified Studies

Four studies met the inclusion criteria but were excluded from quantitative pooling due to unavailable or incompatible survival data stratified by timing (Djamel-Eddine 2019 [16], Furtak 2022 [18], Woodroffe 2020 [24], Dirks 1993 [15]).

Djamel-Eddine et al. (2019) reported 54 reoperated patients with a median postoperative survival of 8.9 months and 6% morbidity, but no timing-based comparisons. Furtak et al. (2022) [18] analyzed 61 cases with an average interval of 11 months; functional improvement occurred in 62% (mean ΔKPS + 5). Woodroffe et al. (2020) [24] reported 67 cases with a median OS of 11.1 months and 8% morbidity but lacked variance data for timing. Dirks et al. (1993) [15] presented an early pre-temozolomide series (n = 43) defining recurrence radiologically, limiting comparability.

Across these four studies (n = 225), reoperation was consistently feasible, with morbidity of 6–9% and preservation of neurological function. Although timing effects were not quantifiable, all studies underscored the importance of preoperative performance status and extent of resection as the principal determinants of outcome.

## 4. Discussion

In this meta-analysis of 18 observational studies, including 14 with extractable survival data, earlier reoperation for recurrent glioblastoma was associated with a modest but statistically significant improvement in overall survival compared with delayed reoperation. The pooled hazard ratio of 0.86 indicates an approximate 14% relative reduction in mortality, with low-to-moderate heterogeneity across studies. This benefit appeared most pronounced among patients with good functional status (KPS ≥ 70), those achieving near-total or gross-total resection (EOR ≥ 95%), and those with tumors situated in non-eloquent regions. Importantly, early reoperation did not increase major perioperative morbidity or mortality and was associated with higher rates of postoperative functional stability or improvement.

### 4.1. Comparison with Previous Evidence on Reoperation

Most existing syntheses have focused on whether reoperation itself improves survival rather than the timing of reoperation. Lu et al. [26] pooled more than 1000 patients and found that repeat surgery conferred a survival advantage, though they emphasized the profound effects of selection bias and heterogeneity in surgical indications. Zhao et al. [12] reinforced these findings using time-dependent modeling and demonstrated that reoperation is beneficial when analyzed appropriately; however, their analysis did not differentiate between early and delayed surgical interventions.

Systematic reviews by Montemurro et al. [27] and Soults et al. [28] also concluded that second surgery may prolong survival in selected patients but noted major limitations in reporting, including absence of standardized timing definitions and incomplete adjustment for functional status and EOR. The present analysis extends this body of literature by quantifying timing as a variable of interest and demonstrating that survival benefit is tied to both operative timing and completeness of cytoreduction.

### 4.2. Evidence on Timing and Patient Selection

Few contemporary studies have explicitly evaluated timing. Kalita et al. [8] (2023) observed that earlier reoperation (within ~24 months) correlated with improved survival, particularly in younger, high-functioning patients, while cautioning against overuse of surgery in late, frail recurrences. Lecce et al. [20] proposed a real-world selection algorithm prioritizing KPS ≥ 70, non-eloquent tumors, and acceptable surgical windows, reporting improved outcomes when surgery occurred earlier in the recurrence trajectory. González et al. [19] compared fixed-time versus time-dependent analyses and showed that failure to account for immortal-time bias may inflate the apparent benefit of reoperation.

More equivocal findings have also been reported. Yang et al. [25] found no clear survival difference between reoperated and non-reoperated controls in a matched cohort with heterogeneous timing and many eloquent or multifocal tumors. Honeyman et al. [9] similarly observed no significant difference between early (<6 months) and later reoperation in progressive IDH-wildtype glioblastoma, though their study was underpowered to determine optimal timing.

Taken together, these reports illustrate the complexity of evaluating timing in heterogeneous clinical populations. Our pooled results indicate that earlier reoperation is beneficial, but only when paired with favourable prognostic factors—consistent with broader evidence linking survival to younger age, MGMT methylation, KPS, and maximal safe resection [29].

The timing of reoperation should be interpreted within the context of multimodality treatment strategies, including systemic therapy, re-irradiation, and emerging therapeutic approaches. In this meta-analysis, timing was evaluated as a decision window rather than a surrogate for tumor biology, and its effect likely reflects interactions with treatment sequencing and patient selection.

### 4.3. Functional Outcomes and Morbidity

A key concern regarding early repeat surgery is cumulative neurological morbidity. Earlier institutional series reported substantial complication rates when reoperation was performed in eloquent regions or in medically fragile patients. Sacko et al. [30] described permanent neurological morbidity approaching 30% in a frail recurrent cohort. Wann et al. [31] likewise reported that survival gains may be offset by non-trivial surgical morbidity.

More recent work, however, has shown that modern surgical techniques significantly reduce morbidity. Studies incorporating intraoperative mapping, neuronavigation, and refined selection criteria report major complication rates of 5–10% [32,33]. Our findings are aligned with this contemporary literature: early surgery did not increase morbidity relative to delayed reoperation, and functional status remained stable or improved in approximately two-thirds of cases. This suggests that judiciously selected early reoperation does not inherently elevate surgical risk and may help prevent functional deterioration associated with delaying intervention.

### 4.4. Relationship to Broader Management of Recurrent GBM

Management of recurrent glioblastoma remains heterogeneous, encompassing reoperation, reirradiation, systemic therapy, and combined modalities [33]. Importantly, temporal considerations are already embedded in other treatment domains: for example, ESTRO/EANO reirradiation guidelines recommend a minimum six-month interval and adequate performance status before retreatment [26]. In contrast, major neuro-oncology guidelines (EANO 2021; NCCN 2023) acknowledge reoperation as an option only in very general terms and offer no operational definition for timing [3,11].

Subventricular zone (SVZ) involvement has been increasingly recognized as a marker of more aggressive tumor biology in glioblastoma, with emerging evidence linking SVZ contact to higher rates of non-local recurrence and dissemination. Although recent studies and meta-analytic data support the prognostic relevance of SVZ involvement, this variable was inconsistently reported and heterogeneously defined across the studies included in the present analysis, precluding quantitative evaluation of its impact on outcomes after reoperation [34].

Our findings support incorporating timing into formal decision frameworks. Early reoperation appears advantageous when tumors remain resectable, the patient retains robust functional status, and a meaningful cytoreductive effect is feasible. Conversely, patients with poor KPS, diffuse or eloquent lesions, or limited resection potential derive minimal benefit and may not warrant early intervention. This aligns with modern perspectives that reoperation should be personalized rather than uniformly applied [35].

### 4.5. Limitations

Although this meta-analysis provides new insights into the prognostic relevance of reoperation timing in recurrent glioblastoma, several considerations temper the strength of its conclusions. Because all included studies were observational, decisions regarding early or delayed reoperation were shaped by clinical judgment, patient preference, and tumor characteristics, which inherently introduce selection effects. Patients offered earlier surgery often had better functional status or more surgically accessible recurrences, factors that independently influence survival. Additionally, definitions of timing varied substantially across studies, making it necessary to harmonize heterogeneous thresholds into broader categories that may blur more subtle effects of surgical interval. Only a minority of cohorts used time-dependent survival methods, which means some degree of immortal-time bias cannot be excluded despite sensitivity analyses. Reporting of postoperative neurological outcomes, quality of life, and complications was inconsistent, limiting the precision with which functional consequences of surgical timing could be evaluated. Finally, molecular and volumetric data—now central to glioblastoma prognostication—were available in only a subset of studies.

All included studies restricted their cohorts to adult patients (≥18 years). Although age was commonly included as a prognostic covariate in individual studies, age-stratified outcome data were not reported in a sufficiently consistent manner to allow pooled subgroup or interaction analyses.

A further limitation concerns the interpretation of surgical timing. In this analysis, “early” versus “delayed” reoperation was evaluated strictly as a temporal decision window, based on study-defined intervals, and not as a proxy for tumor aggressiveness, biological behavior, or molecular characteristics. The study was not designed to disentangle timing from underlying tumor kinetics, genetics, or surveillance intensity, as such data were inconsistently reported and not analytically comparable across cohorts. Accordingly, the findings reflect differences in when reoperation was undertaken, rather than the biological determinants of recurrence, and should be interpreted within this methodological framework.

This limitation underscores that the present findings address when reoperation occurs, not why recurrence evolves at a given pace, and reinforces the need for future prospective studies integrating standardized timing definitions with molecular and imaging-based markers of tumor behavior.

Finally, observational data cannot fully disentangle the effect of surgical timing from tumor biology and patient selection, and causal inference remains limited despite time-aware modeling and sensitivity analyses.

These limitations highlight the need for prospective, standardized research to refine the evidence base around the timing of repeat surgery.

## 5. Conclusions

This meta-analysis demonstrates that earlier reoperation for recurrent glioblastoma is associated with improved survival without increasing major surgical morbidity, particularly among well-selected patients with good functional status, resectable non-eloquent recurrence, and a realistic prospect of achieving near-total resection. Timing should therefore be considered an integral parameter in multidisciplinary decision-making. Future prospective multicenter research with standardized timing definitions, volumetric analytics, and time-dependent survival modeling is essential to refine evidence-based recommendations and guide individualized care pathways in recurrent glioblastoma.

## Figures and Tables

**Table 1:** Summary of Included Studies Evaluating Early vs. Delayed Reoperation in Recurrent Glioblastoma.

First Author (Year)	Country/Institution	n (Re-op)	Main Findings/Notes
Azoulay (2017) [13]	Canada—McGill University	69	No survival difference overall; combined therapies common.
Bloch (2012) [14]	USA—UCSF	107	EOR at recurrence correlated with OS; timing not analyzed.
Brandes (2016) [6]	Italy—Bologna group	270	Time between surgeries associated with improved OS; selection bias possible.
Chaichana (2013) [4]	USA—Johns Hopkins	168	Multiple resections associated with prolonged OS; selection-driven effect.
Dirks (1993) [15]	Canada—Toronto	43	Classic early series; historical baseline reference.
Djamel-Eddine (2019) [16]	France—multicenter	53	Focal recurrence subgroup benefited most; limited data granularity.
Franceschi (2015) [17]	Italy—Bologna	102	No independent benefit of reoperation after multivariable adjustment.
Furtak (2022) [18]	Poland—single centre	35	KPS and EOR key survival determinants; limited timing data.
González (2022) [19]	Spain—single centre	33	Advocates early reoperation; time-dependent bias discussed.
Honeyman (2024) [9]	UK—single centre	83	Multiple resections correlated with longer OS; selection bias high.
Kalita (2023) [8]	Czech Republic—multicenter	106	Explicit survival threshold (~22–24 mo) showing survival gain with early re-op.
Lecce (2024) [20]	Italy—IRCCS Regina Elena	138	Threshold of ≥9–12 mo between surgeries predicts OS gain.
Mut (2025) [21]	Turkey—multicentre	30	Adjusted analysis shows early re-op improves OS; KPS ≥ 70 strongest modifier.
Neville (2021) [22]	Brazil—Univ. São Paulo	63	Repeated resections feasible; functional status key predictor.
Ortega (2016) [23]	USA—Johns Hopkins	94	Multiresection series; survival improved with gross-total re-op.
Tully [10] (2016)	Australia—Royal Melbourne Hospital	49	Early reoperation linked to survival, but confounded by KPS and tumor site.
Woodroffe (2020) [24]	USA—Univ. Iowa	37	Imaging and KPS predicted OS; re-op effect unclear.
Yang (2022) [25]	Canada—Multicenter	33	Matched re-op vs. no re-op; HR ≈ 0.86 favoring early surgery.

## Data Availability

The original contributions presented in this study are included in the article/Supplementary Material. Further inquiries can be directed to the corresponding author.

## Supplementary Materials

The following supporting information can be downloaded at: https://www.mdpi.com/article/10.3390/medsci14010040/s1, Table S1: ROBINS-I Risk of Bias Assessment.
