# Supplementary material for: Timing and Benefit of Early Versus Delayed Reoperation in Recurrent Glioblastoma: A Systematic Review and Meta-Analysis of Survival and Functional Outcomes
Source: Med Sci (Basel). 2026 Jan 15;14(1):40. doi: 10.3390/medsci14010040 (PMC12821606; doi:10.3390/medsci14010040)
Supplement: Supplementary file 1 [file medsci-14-00040-s001.zip › medsci-4061945-supplementary.pdf]

Supplementary Table 1. ROBINS-I Risk of Bias Assessment

ROBINS-I Domain	Judgment	Rationale (Study-Specific)
**Bias due to confounding**	**Moderate**	All included studies were observational. Major confounders (KPS, age, extent of resection, tumor eloquence) were variably adjusted; however, molecular factors (MGMT, IDH) and tumor volume were inconsistently reported. Confounding by indication remains plausible despite multivariable adjustment in several cohorts.
**Bias in selection of participants**	**Moderate**	Inclusion was based on undergoing reoperation; patients selected for earlier surgery were generally fitter with more favorable tumor characteristics. Selection mechanisms were clinically driven but transparent and consistent within studies.
**Bias in classification of interventions (timing)**	**Low–Moderate**	Timing of reoperation was clearly defined within individual studies, but thresholds varied (6–22 months). Misclassification across early/delayed groups is unlikely within studies but possible across pooled analyses due to harmonization.
**Bias due to deviations from intended interventions**	**Low**	Surgical timing reflected real-world clinical decisions. No evidence of systematic deviations after classification; co-interventions (adjuvant therapy) were balanced or adjusted for when reported.
**Bias due to missing data**	**Low–Moderate**	Survival data were largely complete. Functional and complication outcomes were missing in some cohorts but were not differentially missing by timing group. Sensitivity analyses addressed incomplete reporting.
**Bias in measurement of outcomes**	**Low**	Overall survival was objectively defined. Functional outcomes (ΔKPS, discharge home) were standard clinical measures, assessed similarly across groups without knowledge of future outcomes.
**Bias in selection of the reported result**	**Low**	Outcomes were prespecified (OS, function, morbidity). No evidence of selective reporting; analyses followed PRISMA/MOOSE guidance and were consistent with stated aims.
**Bias due to time-related issues (immortal-time bias)**	**Moderate**	Only a minority of studies used time-dependent Cox or landmark analyses. Fixed-time models risk immortal-time bias; however, sensitivity analyses excluding such studies showed consistent results.
**Overall ROBINS-I judgment**	**Moderate risk of bias**	The evidence base is limited by observational design and residual confounding, but consistency of effect, robustness in sensitivity analyses, and objective survival endpoints support moderate confidence in estimates.
